# Anthropogenic threats to the Vulnerable Andean Condor in northern South America

**DOI:** 10.1371/journal.pone.0278331

**Published:** 2022-12-01

**Authors:** Juan Sebastián Restrepo-Cardona, María Alejandra Parrado, Félix Hernán Vargas, Sebastián Kohn, Fausto Sáenz-Jiménez, Yann Potaufeu, Fabricio Narváez

**Affiliations:** 1 Fundación Cóndor Andino Ecuador, Quito, Ecuador; 2 Department of Wildlife Ecology and Conservation, University of Florida, Gainesville, FL, United States of America; 3 Fundación Neotropical, Bogotá, Colombia; 4 The Peregrine Fund, Galápagos, Ecuador; 5 Escuela de Biología, Universidad industrial de Santander, Bucaramanga, Colombia; 6 Fundación Galo Plaza Lasso, Zuleta, Ecuador; INIBIOMA (Universidad Nacional del Comahue-CONICET), ARGENTINA

## Abstract

Vultures comprise one of the most threatened groups of birds worldwide. With a total population not exceeding 6700 mature individuals, and in rapid decline across its range, the Andean Condor (*Vultur gryphus*) is listed as a Vulnerable species in the IUCN red list. Local population extinctions and decline are of particular concern in northern South America, where no more than 340 condors may exist at present. Despite this, no quantitative assessments exist in Ecuador, Colombia, and Venezuela regarding the threats affecting Andean Condor populations. To address this, we compiled records of Andean Condors injured, or killed, between 1979 and 2021. We obtained data of 164 condors affected by different causes of injury, of which 83.5% were reported in Ecuador, 15.2% in Colombia, and 1.2% in Venezuela. Of the total number, 84.7% of the injured individuals died. Between 1979 and 2021, in Ecuador, Colombia and Venezuela, at least 103 Andean Condors were presumably poisoned, 22 were shot, and 39 individuals were affected by other causes. The total number of individuals affected by different causes represents between 48% and 72% of the total population estimated in northern South America. Of great concern is the fact that, between 2007 and 2021, poisoning and shooting together caused the loss of 19–31% of the estimated population of condors in Ecuador, and 7–21% of the estimated population in Colombia. Given the important mortality induced by humans, environmental education programs, socio-ecological research, application of environmental laws, and management strategies based on scientific evidence to prevent and mitigate human-wildlife conflicts are urgently required for effective Andean Condor conservation in northern South America.

## Introduction

Human-induced species extinctions over the last 500 years are comparable, both in rate and magnitude, to the main five mass extinctions in Earth´s history [1]. Nearly half of the planet’s bird species are in decline, with one in eight species under threat of extinction [2]. Raptors are one of the most threatened groups of birds worldwide, with vultures being particularly vulnerable [3–5]. Among the 16 species of Old World vultures (Accipitridae), 12 are categorized as Endangered or Critically Endangered, and more than 80% of the species are currently in decline [3]. In the case of the seven species of New World vultures (Cathartidae), 43% are in decline, and the Andean (*Vultur gryphus*) and California Condor (*Gymnogyps californianus*) are categorized as Vulnerable and Critically Endangered, respectively [4]. Despite the increasing concern regarding the conservation status of New World vultures, there is little information available on the factors that threaten them [4–6].

The Andean Condor inhabits mountain grasslands along the Andes from western Venezuela to southern Argentina and Chile, as well as the Sierra Nevada de Santa Marta in Colombia [7, 8]. As other obligate scavengers, this species provides a key ecosystem service by accelerating carcass decay and reducing the probability of pathogen microorganism transmission to the environment [9, 10]. The Andean Condor is also a source of fascination for humans and an important cultural icon throughout its geographical range [11]. Despite its ecological and cultural importance, the total population of this species probably does not exceed 6700 mature individuals, with an alarming decline reported across its distribution [12]. Local population extinctions and decline are of particular concern in northern South America (Ecuador, Colombia, and Venezuela), where no more than 340 condors may exist at present [13–15].

Several threats affect the Andean Condor throughout its distribution. Poisoning with pesticides, probably the most relevant threat for this species, is due to conflict among humans, carnivores (e.g. domestic dogs *Canis lupus familiaris* and pumas *Puma concolor*), as well as other scavenging birds [6, 16]. Other anthropogenic threats affecting this species are shooting, lead contamination, electrocution, and the use of condors in traditional celebrations [17–20]. Despite the increase in available scientific information pertaining to the Andean Condor, few studies have addressed the factors that threaten this species, and the impacts of these threats remain uncertain in some regions of its distribution, such as northern South America [6]. Indeed, in this geographical region, there have been no quantitative assessments conducted of the threats affecting Andean Condor populations. Despite the fact that recent information suggests poisoning as the main cause of condor death in Ecuador and Colombia [6], the magnitude of this conservation problem is still unknown.

Direct threat analysis is a valuable tool in biodiversity conservation, particularly for threatened species with negative population trends [21]. Quantitative examination of threats to the Andean Condor in northern South America is key to support evidence-based decisions for the conservation of this species. The aim of this study is, therefore, to assess, for the first time, records of Andean Condors injured in Ecuador, Colombia, and Venezuela between 1979 and 2021 to better plan conservation actions for the species in this geographical region.

### Study area

Our analysis was focused on the Andean Condor threats in northern South America. In Ecuador, the Andean Condor is distributed throughout the Andes, from the border with Peru to that with Colombia. Anecdotal accounts by Humboldt and Whimper in 1802, as well as by Wiggins in 1945, provide observations of more than 100 condors at different Ecuadorian localities [22]. In contrast, the results of two nationwide censuses conducted in 2015 and 2018 indicate a total population ranging between 94 and 150 individuals [14, 15].

In Colombia, the historical distribution of the condor included the Andes, the Serranía del Perijá and the Sierra Nevada de Santa Marta. Due to anthropogenic threats, the wild populations had decreased by the late 1980s, and they are currently restricted to the northeastern and southern regions of the country [13, 23]. Estimates suggest that the Andean Condor population in Colombia ranges from 135 to 190 individuals [13], although this estimate is not supported by quantitative data obtained from field sampling.

In Venezuela, the historic distribution of the species was described from eight records in the Merida Cordillera and Sierra de Perijá. However, it is considered that these condors may actually have been from Colombia [24, 25]. Its resident species status has been questioned by the Venezuelan ornithologist community, and the Andean Condor is likely a transient visitor in this country, since there is no evidence of its natural history, breeding biology, or any archaeological or anthropological records showing otherwise [24].

## Materials and methods

In order to understand the threats faced by Andean Condors in Ecuador, Colombia, and Venezuela, we compiled injury and death records of wild-born, released, and rescued individuals between 1979 and 2021. To determine the cause of injury of the individuals analyzed, we obtained information from veterinary diagnostic reports, necropsies, x-rays, lead concentration examinations, and pesticide analysis. Moreover, we interviewed Andean Condor specialists in order to complement these records, avoiding counting the same case twice.

Data pertaining to affected condors in Colombia was obtained from Natural National Parks of Colombia, as well as wildlife rescue centers in the following regional public offices: CAS in Santander, CORPONOR in Norte de Santander, and CORPOBOYACÁ in Boyacá. Data on affected individuals in Ecuador was obtained from Parque Cóndor, the Galo Plaza Lasso Foundation, the Ilitio Rescue Center, the Andean Condor Foundation, the National Andean Condor Working Group, and the Planeta y Vida Veterinary Clinic, as well as from the Quito and San Martin Zoos. Data pertaining to affected individuals in Venezuela was obtained from the Museo Estación Biológica de Rancho Grande.

We also collected information from specimen records in biological collections. Records from specimens in Colombia came from the Museo de Historia Natural de la Pontificia Universidad Javeriana, and the Museo de Historia Natural de la Universidad de Caldas. Specimen records in Ecuador come from the Museo de Ciencias Naturales del Colegio San José la Salle, Museo de Ciencias Naturales Gustavo Orcés, Museo Etnográfico del Colegio Nacional Mejía, the Instituto Nacional de Biodiversidad, and the Unidad de Protección de Medio Ambiente de la Policia Nacional.

### Data analysis

We used Chi-square independence tests to assess whether significant differences existed among the number of condors affected by each threat regarding the total number of condors affected by the other causes of injury. These tests were performed using R software version 2.1 [26]. We considered the results to be statistically significant when p < 0.05.

## Results

We obtained records of 164 Andean Condors affected by different causes of injury between 1979 and 2021. The age and sex of the injured condors were undetermined in the vast majority of the reports; however, in the cases where these parameters were determined, there was a higher number of injured adults than immature individuals, and a higher number of injured males than females (Table 1). Of the total number of records, 83.5% were reported in Ecuador, 15.2% in Colombia, and 1.2% in Venezuela. Captive-born condors comprised 17 records, of which 12 were from Colombia, three from Ecuador, and two from Venezuela. Rescued individuals accounted for 25 records, of which nine were released following rehabilitation, while the other 16 remained in captivity. A total of 84.7% of the condors we recorded died (Table 1).

**Table 1:** Number of Andean Condors affected (percentage values in parenthesis), and number of events by cause of injury, age (A = adult, I = immature, U = unknown), sex (M = male, F = female, U = unknown), individual birth condition (Wb = wild-born, Cb = captive-born), country (E = Ecuador, C = Colombia, V = Venezuela), and individual status (D = dead, Rc = recovered and in captivity, Rf = recovered and free-ranging), in northern South America between 1979 and 2021.

Causes of injury	Number of individuals affected	Number of events	Age			Sex			Condition		Country			Status		
			A	I	U	M	F	U	Wb	Cb	E	C	V	D	Rc	Rf
**Poisoning**	103 (63%)	25	18	6	79	17	8	78	100	3	90	13		97	1	5
**Shooting**	22 (13%)	22	9	9	4	13	7	2	19	3	18	2	2	13	8	1
**Illegal trafficking**	10 (6%)	1			10			10	10		10			10		
**Trauma**	5 (3%)	5		5		4	1		5		5				2	3
**Electrocution**	3 (2%)	3		2	1	2		1	2	1	1	2		3		
**Falling from nest**	2 (1.2%)	2		2		1	1		2		1	1		1	1	
**Starvation**	2 (1.2%)	2		2		1	1			2	2			2		
**Illegal capture**	2 (1.2%)	2		2		2			2		2				2	
**Collision with vehicle**	1 (0.6%)	1			1			1	1		1			1		
**Free-ranging dogs attack**	1 (0.6%)	1		1		1			1		1				1	
**Gastrointestinal infection**	1 (0.6%)	1		1		1				1		1		1		
**Undetermined**	12 (7.3%)	12	1	8	3	5	5	2	5	7	6	6		11	1	
**Total**	164 (100%)	77	28	38	98	47	23	94	147	17	137	25	2	139	16	9

The most important threat to the Andean Condor was poisoning (X^2^ = 10.7, df = 1, p < 0.05), followed by shooting. In addition, 10 condors were illegally trafficked, five suffered from trauma, three were electrocuted on high-tension wires, two fell from the nest, two were illegally kept in captivity, two died of starvation, one collided with a vehicle, one died of a gastrointestinal infection, and one hatchling was attacked by free-ranging dogs. The cause of injury could not be determined for 12 condors (Table 1).

Poisoning events involved groups of between 1 and 30 condors, whereas those related to other causes only affected a single individual in each case. In an illegal trafficking event reported in 1988 in Ecuador, 10 condors were captured in order to be sent abroad. The chemical compounds used for poisoning Andean Condors were organophosphates, sodium monofluoroacetate, and carbofuran. In addition, anticoagulants (warfarin) and pyrethrins were also detected. Fourteen condors showed lead concentrations of between 2 ug/dL and 31 ug/dL (Table 1, S1 Table).

**Fig 1:**
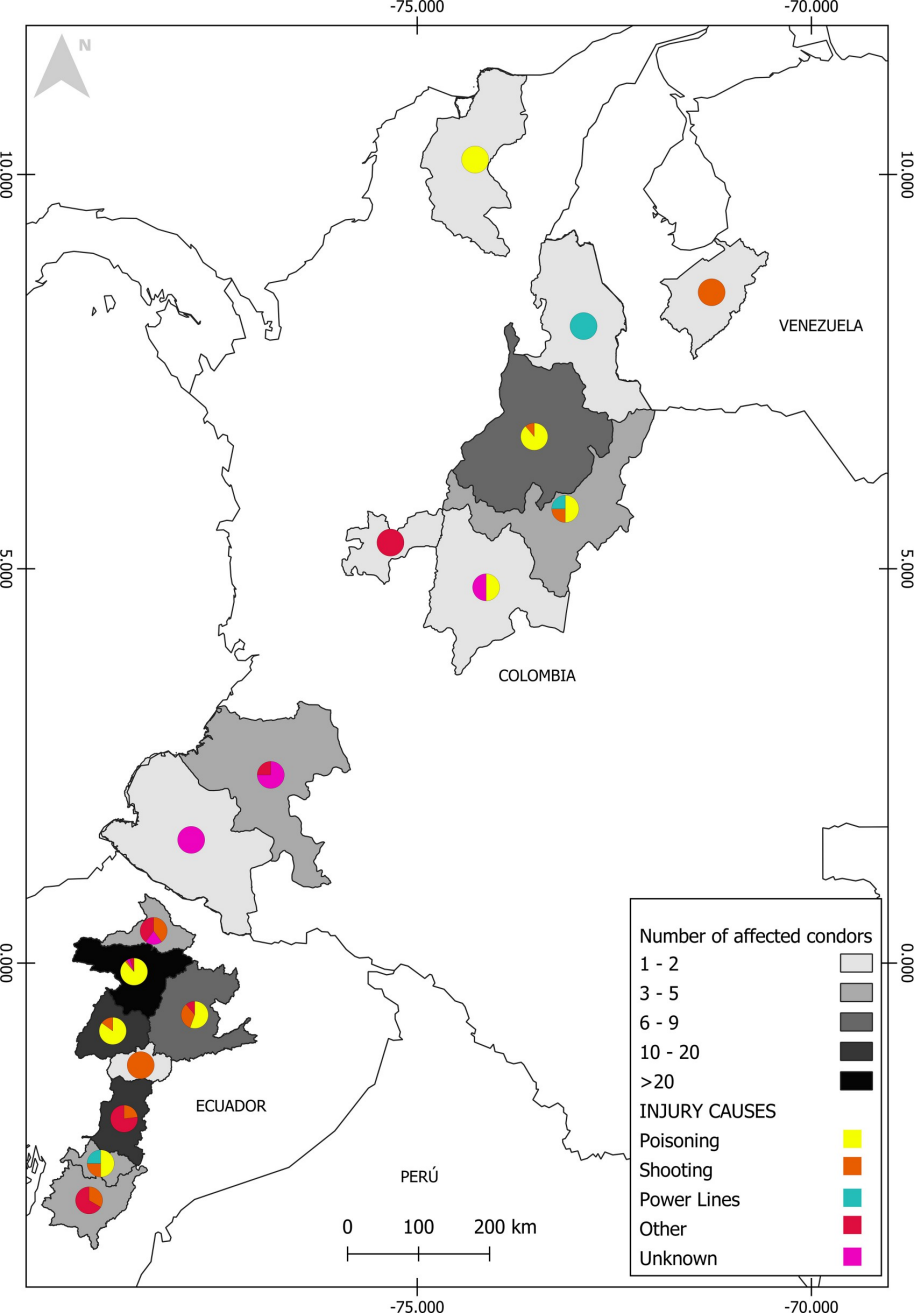
Number of Andean Condors affected by poisoning, shooting, and other causes of injury in northern South America between 1979 and 2021. Reprinted from http://creativecommons.org/licenses/by/4.0/ under a CC BY license, with permission from Emily Chenette, original copyright 2022.

In Ecuador, 108 records of poisoned or shot condors originated in eight provinces, mainly Pichincha (72 cases), followed by Cotopaxi (20 cases), Napo (6 cases), Imbabura (4 cases), and Cañar (2 cases). The provinces of Chimborazo, Tungurahua, and Azuay only presented a single case each and, for one individual, it was not possible to determine the province of origin. In Colombia, there were 15 records of poisoned or shot condors, distributed in four departments. Santander has the largest number of records, with nine cases, followed by Boyacá (three cases), Magdalena (two cases), and Cundinamarca (one case). In Venezuela, the two records were both from Sierra de La Culata National Park (Fig 1, S1 Table).

## Discussion

Our results suggest that, in northern South America, poisoning is the main cause of injury and death for the Andean Condor, followed by shooting, accounting for 63% and 13% of cases, respectively. Condors also face other anthropogenic threats, including electrocution, illegal capture and trafficking, collision with vehicles, and free-ranging dogs attacks, all of which were previously unknown to affect condor populations in the northern Andes. Between 1979 and 2021, in Ecuador, Colombia and Venezuela, at least 125 Andean Condors were poisoned or shot, while 39 individuals were affected by other causes (Table 1). The total number of individuals affected by different causes represents between 48% and 72% of the total population in northern South America, which has been estimated to be between 229 and 340 individuals [13–15].

Our study adds evidence indicating that poisoning is the most important threat to the Andean Condor throughout its distribution. In Ecuador and Colombia, 103 condors were poisoned between 1979 and 2021, of which only six individuals could be rescued, and five were subsequently rehabilitated and released (Table 1). In Peru, Bolivia, Argentina and Chile, at least 174 individuals were affected by this threat between 1993 and 2021 [18–20, 27]. The use of organophosphates and carbamates on carrion has been reported as a relevant threat to the Andean Condor [19], but our record of anticoagulants and pyrethrins in condors is the first reported to the species [28].

Based on the information provided by Andean Condor specialists, we found that at least 16% of the condors killed in Ecuador consumed carrion which had been deliberately poisoned in response to cattle loss caused by free-ranging dogs (S1 Table). In Colombia, the evidence indicates that conflict among rural inhabitants, carnivores, and condors could have triggered human attacks towards the Andean Condor [29]. For effective management of the Andean Condor, it is therefore important to assess the impact of free-ranging dogs on condor populations in northern South America, either through direct attacks on individuals (one reported case is included in this study), competition for food, or by causing conflict with rural inhabitants, which leads in turn to unintentional subsequent poisoning of condors.

Shooting is another important threat to the Andean Condor in northern South America. Although nine of 22 shot condors were rescued, only one individual was subsequently rehabilitated and released (Table 1). The available information on Andean Condor hunting is scarce. A recent study indicates that four condors were affected by this threat in Peru [20]. In Chile, 18 individuals entering a rehabilitation center had fragments of ammunition in their bodies [18]. Application of the law is key to stop Andean Condor population declines [6]. In 2013, a person was sentenced in Ecuador for being responsible for shooting and killing a juvenile condor in the province of Azuay, thus setting a precedent for the application of environmental law in the region. Shooting may not only be a direct cause of individual death, but could also contribute to the accumulation of lead in blood and other tissues [6].

Lead contamination is a major threat to Andean Condors throughout their range [6, 17]. Our data show that lead concentrations in condor blood in Ecuador and Colombia ranged from 2 μg/dL to 31 μg/dL (S1 Table). In four condors, we found that these concentrations exceeded the sublethal threshold of 20 μg/dL [17]. Deaths associated with lead contamination are often isolated rather than in massive events, so this impact could be hidden and remain undiagnosed in most sites where the Andean Condor occurs [6]. In North America, the California Condor came close to extinction during the 1980s, mainly as a result of lead contamination from feeding on game animals containing bullet fragments [30]. Replacement of lead with non-toxic ammunition may therefore make a useful contribution to the conservation of the Andean Condor in the countries it inhabits [31].

Andean Condor reintroduction efforts were carried out between 1989 and 2013 in Colombia, between 1993 and 2001 in Venezuela, and during 2016 in Ecuador. In these efforts, 71, 13 and three captive-born individuals were released in these countries, respectively [23, 25, 32] (S1 Table). We found that at least 17% of the condors released in Colombia, and 15% of those released in Venezuela, were affected by anthropogenic causes. Among these individuals, three were poisoned, three were shot, and one was electrocuted on high-tension wires. In Ecuador, two condors had to be captured a few days following released, due to starvation, and these individuals subsequently died in captivity (Table 1, S1 Table). Due to the lack of monitoring following condor releases in northern South America, little is known regarding the threats to the species at its repopulation sites. For the success of Andean Condor release programs, it is essential to include the elimination of threats affecting the survival of the species in the wild. The reintroduction program of the California Condor has to date been a success due to the implementation of threat reduction actions in the wild, at the same time as captive breeding and release efforts [33]. In this sense, our results provide relevant information to better plan reintroduction actions taking in consideration threats affecting Andean Condors in northern South America.

We acknowledge that our study has some limitations. For instance, data on key parameters such as age and sex were not collected systematically, particularly in the early years of this study. We also acknowledge that it was not always possible to identify the type of poison used to injure or kill condors because of the limited facilities with which to conduct this type of laboratory analysis in Ecuador and Colombia. Considering the protected status of the Andean Condor, it is also possible that a number of condors, in addition to those reported here, could have been persecuted but not reported in order to avoid legislative sanctions. Despite these limitations, we believe that our analysis provides useful information on the relative importance of different threats affecting the species in northern South America.

The persistence of many scavenging bird populations will undoubtedly depend upon human ability to recognize and take action to mitigate threats [10]. Of great concern is the fact that, between 2007 and 2021, poisoning and shooting together caused the loss of 19–31% of the estimated population of condors in Ecuador, and 7–21% of the estimated population in Colombia. As our results indicate, these two threats affected Andean Condor populations across much of their distribution in northern South America, including eight provinces of Ecuador, four departments of Colombia, and a National Park in Venezuela (Fig 1), making this a regional conservation issue. Elimination of Andean Condor poisoning, particularly in Pichincha, Cotopaxi, and Santander where 92% of the total condor poisoning cases were reported, is a challenging goal. Accomplishing this task requires the identification and monitoring of areas of high poisoning risk, development of environmental education programs and socio-ecological research, application of environmental laws, and implementation of management strategies based on scientific evidence to prevent and mitigate human-wildlife conflicts.

## Supporting information

S1 Table All data obtained of 164 Andean Condors affected by different causes of injury in Ecuador, Colombia and Venezuela, between 1979 and 2021. (XLSX)
